# GP working time and supply, and patient demand in England in 2015–2022: a retrospective study

**DOI:** 10.3399/BJGP.2024.0075

**Published:** 2024-09-17

**Authors:** Rosa Parisi, Yiu-Shing Lau, Peter Bower, Katherine Checkland, Jill Rubery, Matthew Sutton, Sally Giles, Aneez Esmail, Sharon Spooner, Evangelos Kontopantelis

**Affiliations:** Division of Informatics, Imaging & Data Sciences, School of Health Sciences, University of Manchester, Manchester.; Health Organisation, Policy and Economics (HOPE) Group, Centre for Primary Care and Health Services Research, University of Manchester, Manchester.; Division of Population Health, Health Services Research and Primary Care, University of Manchester, Manchester.; Health Organisation, Policy and Economics (HOPE) Group, Centre for Primary Care and Health Services Research, University of Manchester, Manchester.; Alliance Manchester Business School, University of Manchester, Manchester.; Health Organisation, Policy and Economics (HOPE) Group, Centre for Primary Care and Health Services Research, University of Manchester, Manchester.; NIHR Greater Manchester Patient Safety Translational Research Centre, University of Manchester, Manchester.; Division of Population Health, Health Services Research and Primary Care, University of Manchester, Manchester.; Health Organisation, Policy and Economics (HOPE) Group, Centre for Primary Care and Health Services Research, University of Manchester, Manchester.; Division of Informatics, Imaging and Data Sciences; NIHR School for Primary Care Research, Division of Population Health, Health Services Research and Primary Care, School of Health Sciences, University of Manchester, Manchester.

**Keywords:** general practice, health services research, primary care, workforce

## Abstract

**Background:**

English primary care faces a reduction in GP supply and increased demand.

**Aim:**

To explore trends in GP working time and supply, accounting for factors influencing demand for services.

**Design and setting:**

Retrospective observational study in English primary care between 2015 and 2022.

**Method:**

Trends in median GP contracted time commitment were calculated using annual workforce datasets. Three measures of demand were calculated at practice-level: numbers of patients; numbers of older patients (≥65 years); and numbers of chronic conditions using 21 Quality and Outcomes Framework disease registers. Multi-level Poisson models were used to assess associations between GP supply and practice demand, adjusted for deprivation, region, and year.

**Results:**

Between 2015 and 2022, the median full-time equivalent (FTE) of a fully qualified GP decreased from 0.80 to 0.69**.** There was a 9% increase in registered population per GP FTE (incidence rate ratio [IRR] = 1.09; 95% confidence interval [CI] = 1.05 to 1.14). This increase was steeper using numbers of chronic conditions (32%, IRR = 1.32; 95% CI = 1.26 to 1.38). Practices in the most deprived decile had 17% more patients (IRR = 1.17; 95% CI = 1.08 to 1.27) and 19% more chronic conditions (IRR = 1.19; 95% CI = 1.06 to 1.33) per GP FTE, compared with the least deprived decile. These disparities persisted over time. All regions reported more chronic conditions per GP FTE than London.

**Conclusion:**

Population demand per GP has increased, particularly in terms of chronic conditions. This increase is driven by several factors, including a reduction in GP contracted time commitments. Persistent deprivation gradients in GP supply highlight the need to recruit and retain GPs more equitably.

## Introduction

Primary care occupies a foundational role in the healthcare system in England, delivering health care for most chronic and acute conditions, and promoting the health of the population. Ensuring adequate numbers of GPs is one of the biggest challenges for healthcare planners. The NHS in England is facing increasing and ongoing reduction of the primary care workforce, particularly of GPs.^1^^–^3 The decrease in GP supply is a result of several factors such as early retirement,^4^^,^^5^ reduction in working hours or leaving the profession,^6^ high levels of GP turnover and low retention,^7^ insufficient number of newly trained GPs joining the workforce, and a lack of overseas recruitment.^8^ Studies have also shown links between GP supply and adverse health outcomes, such as mortality,^9^^,^^10^ hospital visits and attendances,^11^^,^^12^ self-reported health,^13^ continuity of care,^14^ and quality of care.^15^

In the context of an increasingly ageing population with more complex needs, these issues lead to an increasing mismatch between workload and workforce funding, and have widened the gap between the supply of healthcare staff and their availability.^16^^,^^17^ In 2015, the government promised 5000 more GPs by 2020 and, in 2019, an additional 6000 GPs by 2024.^18^ These promises have not been met and, recently, the Health Foundation projected a shortfall of GP supply–demand of 18 900 full-time-equivalent (FTE) by 2030/2031.^19^

The reduction in GPs began before the COVID-19 pandemic, but the pandemic has exacerbated the disparity between supply and availability.^20^ Although the primary care workforce crisis is a national challenge for England and the wider UK, several studies have reported that the challenges are greater in socially deprived areas.^17^^,^^21^^,^^22^ While populations in these areas tend to have higher morbidity and complex health needs, they are usually served by fewer GPs.^8^^,^^16^^,^^23^^,^^24^

Existing studies have reported on the numbers and FTE of GPs over time,^1^^–^3^,^^17^ examined the inequalities in the distribution of the primary care workforce in England, and the relationship with access or quality of care.^13^^,^^15^^,^^17^^,^^21^ However, up-to-date, post-pandemic information is needed in the context of regional and deprivation-driven inequalities. Previous work has focused on unadjusted practice population registers with regional variation in inflation levels and the highest levels observed for London.^25^ More importantly, previous estimates^17^^,^^21^ have not accounted for the changing health needs of the population, although some work has attempted to address that by comparing GP supply to older populations only.^1^^,^^26^^,^^27^

**Table:** How this fits in

The NHS in England is facing an increasing reduction of the primary care workforce, particularly of GPs. Existing studies have reported on numbers of GPs over time and found that GP supply is less in socially deprived areas. However, up-to-date, post-pandemic information is needed as a result of widening regional and deprivation-driven inequalities. Importantly, previous work has not accounted for the changing health needs of the population. The findings of this study show that, during the study window from 2015 to 2022, GP supply has decreased by 2.7%, while there has also been an increase in demand, with the size of practice populations increasing by 9% and the numbers of patients with chronic conditions increasing by 32%. The reduction in GP supply can be attributed to the reduction of the time GPs are contracted to work (a decrease of 8.7% average FTE), although GP head counts have increased by 5.9%. These findings suggest that, in addition to policies aimed to recruit and retain more GPs, it is also necessary to have policies that incentivise GPs to work and remain in deprived areas, towards more equitable levels of care.

This study explores the distribution of GP supply in England over time, and its regional and socioeconomic variation, adjusted for census population and number of chronic conditions. More specifically, the aim of the study was to quantify contracted time commitment of GPs over time; to calculate three measures of demand at practice-level (numbers of patients; numbers of older patients (≥65 years); and numbers of chronic conditions using 21 Quality and Outcomes Framework [QOF] disease registers); and to explore variation in these estimates, by region and socioeconomic deprivation.

## Method

The study follows the Strengthening the Reporting of Observational Studies in Epidemiology (STROBE) guidelines.^28^

### Data sources and study design

The study used a retrospective observational design for the years 2015–2022, combining data from the GP workforce^29^ and other datasets including information on social deprivation,^30^ the number of chronic conditions of a practice,^31^ and census population^32^^,^^33^ (see Supplementary File page 3 section ‘Data Sources’ for details). The total GP head count and FTEs, practice characteristics, and the individual-level FTE of GPs were extracted from the GP workforce data. This information is included in two independent datasets, which are not linked.

GP supply was explored using the following measures: the number of patients registered with the practice per GP FTE; the number of older patients (≥65 years) registered with the practice per GP FTE; and the number of chronic conditions recorded in 21 QOF clinical registers per GP FTE. The total number of patients (all or older patients only) in the practice or on the QOF register were weighted by the census population because of over-registration of practices (see Supplementary File page 4 section ‘Weighting for the population Census’ for details).^25^ Only fully qualified GPs were included in the analysis.

### Statistical analyses

GP records were excluded if their FTEs were greater than 2.3^34^ or if practices had fewer than 750 patients or other quality issues (total number of patients, or total FTEs excluding trainees = 0 or QOF register = 0) (2775 in total, or 5.1%).

Descriptive statistics (median, first, and third quartiles) and box–whiskers plots were used to summarise the distribution of: individual FTE of GPs by year, sex, regions, and integrated care boards (ICBs); the number of patients (overall or older patients) in the practice; or the number of chronic conditions on the QOF register per GP FTE by year, social deprivation, regions, and ICBs.

Three multivariable multi-level Poisson models were used with random effects to assess the association between practice and population characteristics (independent variables) and the number of registered patients in a practice over GP FTE; or the number of older registered patients in a practice over GP FTE; or the number of chronic conditions on the QOF clinical registers over GP FTE (exposure or dependent variables). Independent variables in the models included social deprivation captured by the Index of Multiple Deprivation (IMD) 2015 in deciles, seven English regions, and year. ICBs were included in the model as random effects.

For each model, interactions between year and social deprivation, or year and regions, were tested. After exclusion criteria were applied, there were no missing values in the variables included in the statistical models.

A sensitivity analysis was performed where the number of patients with diabetes on the QOF register (rather than all the conditions on the QOF register) per GP was included as a dependent variable. The reason for this was that patients on the QOF registers can be counted more than once if they have multiple conditions. All analyses were performed in Stata (version 17).

For the GP supply and three outcomes of interest, log percentage^35^ was used to decompose: the change in FTE GP supply due to change in the GP head counts and average FTE; and the increase in patients’ health demand relative to FTE GP supply (see Supplementary File pages 26–27 section ‘Log percentage decomposition’ for the formulae used).

## Results

When exploring patterns of GPs’ working time during the study window, information included the FTE of 306 601 GPs between 2015 and 2022. During this time window, the median contracted FTE for each fully qualified GP decreased from 0.80 to 0.69 (Table 1) (see Supplementary Figure S1 for details). This decrease was mainly due to a reduction of contracted time among males (median 0.99 to 0.85 FTE) rather than females (median 0.67 to 0.66 FTE) (Table 1) (see Supplementary Figure S2 for details). Overall, the lowest contracted FTE per GP was in the region of London; however, the South West and South East had the largest decrease in contracted FTE per GP (from median FTE of 0.80 to 0.67) (see Supplementary Table S1 and Supplementary Figure S3 for details).

**Table 1.** Distribution of contracted full-time equivalent (FTE) for each fully qualified GP

**Year**	**All GPs**	**Male GPs**	**Female GPs**

	**Median (1st to 3rd quartile)**	**Median (1st to 3rd quartile)**	**Median (1st to 3rd quartile)**
2015	0.80 (0.56 to 1.00)	0.99 (0.72 to 1.07)	0.67 (0.53 to 0.88)
2016	0.80 (0.56 to 1.00)	0.99 (0.72 to 1.07)	0.67 (0.53 to 0.87)
2017	0.78 (0.53 to 1.00)	0.93 (0.67 to 1.00)	0.67 (0.49 to 0.85)
2018	0.75 (0.53 to 1.00)	0.93 (0.67 to 1.01)	0.67 (0.49 to 0.80)
2019	0.75 (0.53 to 0.99)	0.89 (0.64 to 1.00)	0.67 (0.48 to 0.80)
2020	0.72 (0.53 to 0.99)	0.88 (0.64 to 1.00)	0.65 (0.48 to 0.80)
2021	0.70 (0.53 to 0.96)	0.85 (0.64 to 1.00)	0.64 (0.48 to 0.80)
2022	0.69 (0.53 to 0.93)	0.85 (0.64 to 1.00)	0.66 (0.48 to 0.80)

When exploring GP supply, the average number of practices included was 6793 (standard deviation = 361) per year. Between 2015 and 2022, the median (first; third quartile) number of patients in a practice per GP FTE was 2332.1 (1970.5; 2314.4), the number of older patients per GP FTE was 346.3 (239.4; 460.9), and the number of chronic conditions per GP FTE was 1227.1 (949.3; 1604.3) (see Supplementary Tables S2–S4 and Supplementary Figures S4–S6 for details).

During the study window, GP supply decreased by 2.7%. This reduction was a result of the reduction of the time GPs are contracted to work (a decrease of 8.7% in average FTE), although GP head counts increased by 5.9% (see Supplementary File, pages 26–28 for details).

### Statistical analyses

**Figure 1.**
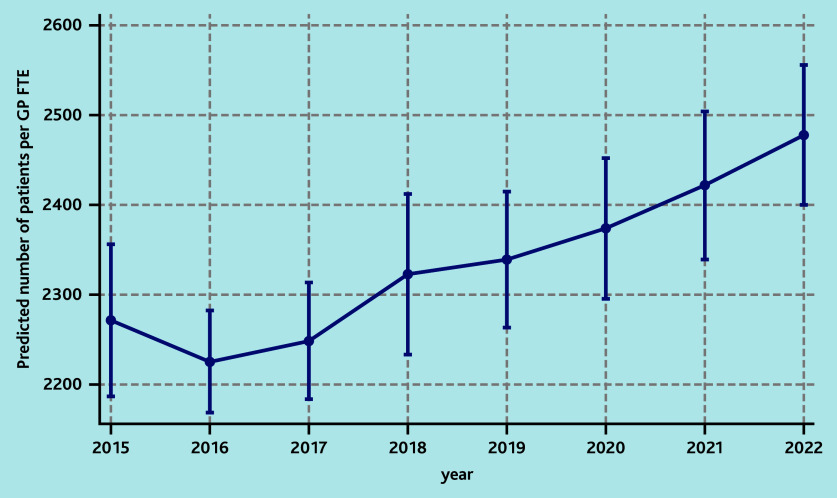
Predicted number of practice patients per GP FTE by year of the fully adjusted multivariable model assessing the association between risk factors and the number of patients in the practice per GP FTE. FTE = full-time equivalent.

In the fully adjusted model, social deprivation (any decile above the fifth) and year were significantly associated with an increase in the number of patients in the practice per GP FTE (Table 3). In particular, the highest decile of deprivation was associated with a 17% increase (incidence rate ratio [IRR] = 1.17; 95% confidence interval [CI] = 1.08 to 1.27) in the number of patients per GP FTE compared with the least deprived area, which corresponded to a predicted number of 2569 patients versus 2194 patients (see Supplementary Figure S7 for details). There was also a significant increasing trend in the number of patients in the practice per GP FTE during the study window, with 9% (IRR = 1.09; 95% CI = 1.05 to 1.14) more patients in the practice per GP FTE in 2022 compared with 2015 (Table 3), which corresponded to a predicted number of 2478 patients versus 2271 (Figure 1).

**Table 3.** Multivariable analyses of the association between risk factors and number of patients in the practice or with number of chronic conditions (patients on the QOF register) or older patients in the practice per GP full-time equivalent

**Variable**	**Practice size**		**QOF all conditions**		**Older patients in the practice**	

	**IRR (95% CI)**	***P*-value**	**IRR (95% CI)**	***P*-value**	**IRR (95% CI)**	***P*-value**
**Social deprivation**						
**IMD 1**	Reference		Reference		Reference	
**IMD 2**	1.02 (0.97 to 1.08)	0.443	1.04 (0.98 to 1.09)	0.183	0.99 (0.94 to 1.05)	0.750
**IMD 3**	1.01 (0.94 to 1.09)	0.751	1.04 (0.95 to 1.14)	0.363	0.95 (0.87 to 1.04)	0.244
**IMD 4**	1.00 (0.95 to 1.06)	0.906	1.04 (0.98 to 1.11)	0.190	0.92 (0.86 to 0.98)	0.009
**IMD 5**	1.07 (1.01 to 1.14)	0.016	1.11 (1.05 to 1.18)	0.000	0.94 (0.87 to 1.02)	0.126
**IMD 6**	1.07 (1.02 to 1.13)	0.006	1.12 (1.06 to 1.19)	0.000	0.90 (0.83 to 0.98)	0.010
**IMD 7**	1.06 (1.01 to 1.10)	0.012	1.10 (1.03 to 1.16)	0.002	0.83 (0.75 to 0.91)	0.000
**IMD 8**	1.08 (1.02 to 1.15)	0.012	1.11 (1.04 to 1.19)	0.001	0.79 (0.72 to 0.87)	0.000
**IMD 9**	1.13 (1.07 to 1.20)	0.000	1.16 (1.09 to 1.23)	0.000	0.78 (0.71 to 0.85)	0.000
**IMD 10**	1.17 (1.08 to 1.27)	0.000	1.19 (1.06 to 1.33)	0.002	0.75 (0.65 to 0.87)	0.000

**Region**						
**East of England**	1.07 (0.96 to 1.19)	0.238	1.38 (1.22 to 1.57)	0.000	1.68 (1.42 to 1.98)	0.000
**London**	Reference		Reference		Reference	
**Midlands**	0.98 (0.91 to 1.05)	0.527	1.36 (1.23 to 1.50)	0.000	1.59 (1.44 to 1.76)	0.000
**North East & Yorkshire &**						
**Humber**	0.99 (0.92 to 1.06)	0.677	1.43 (1.32 to 1.56)	0.000	1.67 (1.51 to 1.84)	0.000
**North West**	1.08 (0.96 to 1.21)	0.191	1.57 (1.34 to 1.84)	0.000	1.78 (1.48 to 2.14)	0.000
**South East**	1.05 (0.95 to 1.16)	0.343	1.31 (1.12 to 1.53)	0.001	1.57 (1.3 to 1.89)	0.000
**South West**	0.90 (0.83 to 0.98)	0.018	1.28 (1.18 to 1.39)	0.000	1.66 (1.52 to 1.82)	0.000

**Year**						
**2015**	Reference		Reference		Reference	
**2016**	0.98 (0.95 to 1.01)	0.177	1.00 (0.96 to 1.03)	0.885	0.98 (0.94 to 1.01)	0.203
**2017**	0.99 (0.96 to 1.02)	0.554	1.04 (1.00 to 1.08)	0.079	0.99 (0.95 to 1.03)	0.681
**2018**	1.02 (0.97 to 1.07)	0.370	1.09 (1.03 to 1.14)	0.002	1.03 (0.98 to 1.09)	0.270
**2019**	1.03 (0.99 to 1.08)	0.194	1.13 (1.08 to 1.18)	0.000	1.04 (0.99 to 1.10)	0.127
**2020**	1.05 (1.00 to 1.09)	0.034	1.19 (1.14 to 1.24)	0.000	1.07 (1.02 to 1.12)	0.010
**2021**	1.07 (1.02 to 1.11)	0.002	1.21 (1.16 to 1.26)	0.000	1.09 (1.04 to 1.14)	0.001
**2022**	1.09 (1.05 to 1.14)	0.000	1.32 (1.26 to 1.38)	0.000	1.12 (1.06 to 1.17)	0.000

*IRR= incidence rate ratio. QOF = Quality and Outcomes Framework.*

In the fully adjusted model, when the outcome was older patients in the practice, practices located in more deprived areas (fourth decile and any decile above the sixth) were associated with a decrease in the number of patients per GP, corresponding to a 25% decrease (IRR = 0.75; 95% CI = 0.65 to 0.87) for the highest decile. Year was significantly associated with a 12% increase of older patients in the practice. The increasing trend of older patients over time was consistent with the trend observed with the general practice population (IRR = 1.12; 95% CI = 1.06 to 1.17) (Table 3).

**Figure 2.**
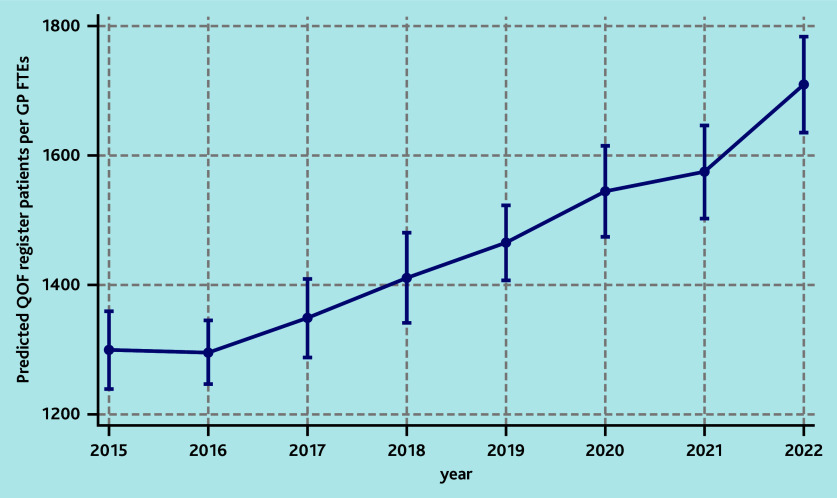
Predicted number of patients with number of chronic conditions per GP FTE by year of the fully adjusted multivariable model assessing the association between risk factors and the number of patients in the practice per GP FTE. FTE = full-time equivalent. QOF = Quality and Outcomes Framework.

Similar or larger associations were obtained when exploring the number of patients on the QOF register per GP FTE. In the adjusted model, practices located in more deprived areas (any decile above the fifth) were associated with an increase in the number of patients with the number of chronic conditions per GP FTE, with the most deprived area associated with a 19% (IRR = 1.19; 95% CI = 1.06 to 1.33) increase compared with the least deprived areas, which corresponded to a predicted number of 1580 patients versus 1328 per GP FTE. An estimate of 32% (IRR = 1.32; 95% CI = 1.26 to 1.38) more patients with the number of chronic conditions per GP FTE in 2022 compared with 2015 corresponded to a predicted number of 1709 versus 1299 patients (Table 3, Figure 2). All regions were associated with more patients with the number of chronic conditions per GP FTE, compared with London. For all models, there was evidence of regional variability during the study window. In particular, South West of England was the region with the lowest number of practice patients per GP FTE between 2016 and 2022 (Table 3) (see also Supplementary Figures S8–S10 for details).

Results were confirmed in the sensitivity analysis, which found that practices located in more deprived areas were associated with an increase in the number of patients with diabetes per GP FTE (see Supplementary Table S8 for details).

When decomposing the log percentage change of the increase in patients’ health demand relative to GP FTEs, the increase was caused by an 8.5% increase in registered patients, or a 28.5% increase in the number of chronic conditions or an 11.8% increase in older patients, respectively; whereas it was caused by a 2.7% decrease in the GP FTEs (total supply) (see Supplementary File, pages 26–28 section ‘Log percentage decomposition’ for details).

## Discussion

### Summary

The findings of this study showed that GP supply has decreased over time during the study period 2015–2022 as a result of both a reduction in the average time that GPs are contracted to work and an increase in the number of practice population or patients with the number of chronic conditions. Specifically, the results show that GPs have reduced their contracted time commitment, between 2015 and 2022, from a median of 0.8 to 0.7 FTE. This trend is mainly a result of male GPs reducing their contracted time commitments. Results showed that the reduction in GP supply was caused by a decrease of 8.7% in average FTE and to a 5.9% increase in GP head counts.

Findings also revealed that the number of patients per GP has increased between 2015 and 2022, and that increase is steeper (32% versus 9%) when taking into account the number of chronic conditions of the population and adjusting for social deprivation and regional variability. The increase in patients’ health demand resulted from an 8.5% increase in registered patients or 28.5% in the number of chronic conditions, and to a 2.7% decrease in GP supply.

**Table 2.** Descriptive statistics on the number of patients, older patients in the practice, or on the Quality and Outcomes Framework (QOF) clinical register per GP full-time equivalent (FTE)

**Year**	**Number of patients in the practice per GP FTE**	**Number of patients ≥65 years in the practice per GP FTE**	**Number of chronic conditions per GP FTE**

	**Median (1st to 3rd quartile)**	**Median (1st to 3rd quartile)**	**Median (1st to 3rd quartile)**
2015	1847.0 (1542.2 to 2277.0)	319.3 (225.2 to 419.9)	1058.1 (836.4 to 1333.6)
2016	1906.7 (1579.2 to 2370.5)	329.8 (228.8 to 434.5)	1114.3 (881.0 to 1409.1)
2017	1935.9 (1594.4 to 2397.9)	336.6 (231.4 to 439.8)	1162.6 (909.4 to 1480.7)
2018	1987.0 (1624.5 to 2502.1)	345.5 (239.8 to 464.1)	1216.5 (935.7 to 1571.7)
2019	2006.8 (1640.7 to 2534.4)	354.1 (245.9 to 476.1)	1268.0 (987.5 to 1654.2)
2020	2025.3 (1648 to 2571.1)	360.4 (250.4 to 482.9)	1329.6 (1034.2 to 1734.7)
2021	2041.3 (1651.0 to 2608.3)	363.6 (251.1 to 487.4)	1342.2 (1050.1 to 1759.4)
2022	2084.6 (1677.0 to 2686.3)	371.0 (259.9 to 503.8)	1449.3 (1126.4 to 1922.9)

Finally, the results highlighted an existing disparity of GP supply between practices located in the least and most deprived areas, when considering practice patients or patients with a number of chronic conditions. However, there was no evidence of these disparities to have become wider during the study window. For older patients the picture was different, with more older patients per GP FTE in the least deprived areas. Regional variation per GP FTE was also observed across the number of patients, the number of older patients, and, especially, the number of chronic conditions, with London being an outlier with the lowest level of demand.

### Strengths and limitations

The study has several strengths. It used national data from England; it has explored for the first time GPs’ working patterns between 2015 and 2022; it provides recent and post-pandemic estimates of GP supply; and it offers more accurate estimates of GP supply by adjusting practice population or those with a number of chronic conditions for the census.

Limitations of the study are as follows. It was not possible to explore trends over time before 2015, because NHS Digital revised the methodology for the GP workforce data after this time. The patterns of GPs’ working time is explored only descriptively, because information on individual-level FTE of GPs is not linked with the practice characteristics. Because the demographic information available in the GP workforce datasets was limited, it was not possible to take more variables into account in the analyses. The analyses include only GPs and do not account for other healthcare professionals; however, new clinical roles have not changed inequalities in the geographical distribution of the primary care workforce^36^ or the quality of care.^37^ Finally, using the QOF to explore the number of chronic conditions is limited by the fact that individuals with multiple long-term conditions are counted multiple times. However, the sensitivity analysis taking into account patients with diabetes on the QOF register per GP confirmed similar trends of the main analysis. It is also worth mentioning that, despite limitations of time and resource, Salisbury *et al* reported that an average of 2.5 problems are discussed in each GP appointment.^38^ It follows that, in any one consultation, GPs and patients with multiple conditions may wish to talk about symptoms or issues related to one or more of their known conditions or discuss new problems or complex health issues that they have. Likewise, current studies have reported variability in the composition of the GP workforce, with London, for example, having the lowest number of practice nurses.^39^

### Comparison with existing literature

This study adds to the literature on the issues of GP supply and the unequal distribution of GPs across socioeconomic strata and regions of England.^40^ The results of the study are in line with most of the literature,^1^^–^3^,^^41^ recording an increasing number of patients per GP FTE.^1^^–^3^,^^24^^,^^41^ However, most of these studies provide only descriptive statistics. The findings of this study are also similar to existing literature highlighting that GP supply is less in most deprived areas.^17^^,^^21^^,^^24^^,^^42^ Fisher *et al*^17^ reported descriptive statistics by defining GP supply in terms of GP workload as in the Carr-Hill formula. Conversely, the increasing trend of patients per GP supply observed in the current study not only takes into account practice characteristics but also the number of chronic conditions of the population. Anecdotally, it has been reported that GPs’ working time has reduced during the study period, which, according to the Health Foundation’s 2023 report,^43^ might be because of greater stress and signs of emotional distress. Nussbaum *et al*^21^ found a decreasing trend of GPs per patient up to 2020 and an unequal distribution of GPs across the least and most deprived decile, with a widening gap over time. However, by using the Carr-Hill formula to calculate the slope index of inequality and measure the difference in FTE GP per patients between the highest and lowest IMD decile, they might have underestimated the reality. The Carr-Hill formula is known to be outdated and does not reflect the population needs for patients living in the most deprived areas.^44^^,^^45^ Finally, similar findings regarding the distribution of FTE GPs by deprivation have recently been found in Scotland,^42^ where the distributions of FTE general practice workforce (including GPs) per 10 000 patients showed fewer FTE clinicians in deprived areas compared with affluent areas. The findings of the current study are in contrast with that of *et al*,^46^ who found a reduction in socioeconomic inequalities in the distribution of GPs between 2004/2005 and 2013/2014. However, comparison with these results is difficult given the different time period analysed and by the revised methodology of the GP workforce data after 2015.

### Implications for practice

The findings of this study show steeper trends in the number of patients in the practice or with the number of chronic conditions per GP FTE compared with previous estimates. The steeper trends are likely because estimates use census-adjusted practice populations and also because patients with a number of chronic conditions have been considered as a denominator to calculate GP supply. The study also found that GP supply is partly driven by the continuous reduction in working time of GPs, an increasing population, and more people with a number of chronic conditions.

Policies are needed to potentially incentivise GPs to work more hours, since the average FTE decrease over time was the largest contributor to the overall decrease in supply. However, this is arguably a systemic problem, with GPs very likely unwilling or unable to face the intense, day-to-day pressures in UK primary care. Therefore, several combined policies are potentially needed, for instance, reducing administrative workload, increasing support by allied healthcare professionals, and incentivising GPs to increase their work hours.^43^ Finally, the study findings also show that the inverse care law^47^ is still very relevant and existing disparities have remained.

The study findings contribute to the literature on the challenges of the primary care workforce and suggest that, in addition to policies aimed to recruit and retain more GPs, it is also necessary to have policies that reduce GP workload, incentivise GPs to increase their work hours, and to work and remain in deprived areas, towards more equitable levels of care.^48^

## Data

Data are publicly available online from NHS Digital, Office for National Statistics, or the Department for Communities and Local Government. The article’s guarantor affirms that the article is an honest, accurate, and transparent account of the study being reported, that no important aspects of the study have been omitted, and that any discrepancy from the study as planned have been explained.
